# Synthesis of SERS-active core–satellite nanoparticles using heterobifunctional PEG linkers†

**DOI:** 10.1039/d1na00676b

**Published:** 2021-11-15

**Authors:** Angela Michelle T. San Juan, Suhash Reddy Chavva, Dandan Tu, Melanie Tircuit, Gerard Coté, Samuel Mabbott

**Affiliations:** Texas A&M University Health Technologies and Innovations Building, 3006 TAMU College Station Texas 77843 USA smabbott@tamu.edu; Department of Biomedical Engineering Emerging Technologies Building 3120 TAMU College Station Texas 77843 USA

## Abstract

Surface-enhanced Raman scattering (SERS) is a sensitive analytical technique capable of magnifying the vibrational intensity of molecules adsorbed onto the surface of metallic nanostructures. Various solution-based SERS-active metallic nanostructures have been designed to generate substantial SERS signal enhancements. However, most of these SERS substrates rely on the chemical aggregation of metallic nanostructures to create strong signals. While this can induce high SERS intensities through plasmonic coupling, most chemically aggregated assemblies suffer from poor signal reproducibility and reduced long-term stability. To overcome these issues, here we report for the first time the synthesis of gold core–satellite nanoparticles (CSNPs) for robust SERS signal generation. The novel CSNP assemblies consist of a 30 nm spherical gold core linked to 18 nm satellite particles *via* linear heterobifunctional thiol–amine terminated PEG chains. We explore the effects that the varying chain lengths have on SERS hot-spot generation, signal reproducibility and long-term activity. The chain length was varied by using PEGs with different molecular weights (1000 Da, 2000 Da, and 3500 Da). The CSNPs were characterized *via* UV-Vis spectrophotometry, transmission electron microscopy (TEM), *ζ*-potential measurements, and lastly SERS measurements. The versatility of the synthesized SERS-active CSNPs was revealed through characterization of optical stability and SERS enhancement at 0, 1, 3, 5, 7 and 14 days.

## Abstract 2

Here we report the synthesis of core–satellite nanoparticles to explore tunable SERS hot-spot generation, signal reproducibility and long-term activity.

## Graphical abstract

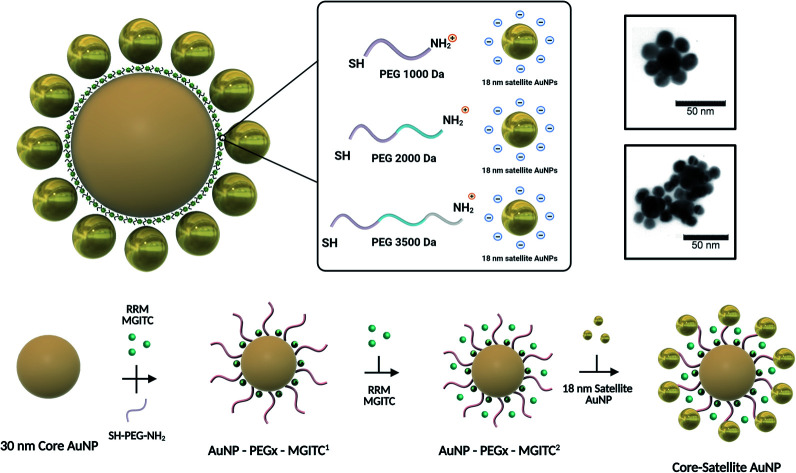

## Introduction

Surface-Enhanced Raman Spectroscopy (SERS) is a versatile analytical technique commonly recognized for its superior sensitivity and ability to generate molecular fingerprints of chemicals that can allow them to be uniquely identified.^1–3^ SERS causes significant signal amplification of target molecules once adsorbed onto a noble metal surface resulting in intensified Raman scattering. The intensity of SERS spectra is several orders of magnitude larger than that of conventional Raman scattering.^1–3^ Over the course of nearly 4 decades since the discovery of SERS, the technique has been applied to a multitude of applications including diagnostic assays,^4,5^ identification of environmental contaminants,^6,7^ and food monitoring applications.^7,8^

SERS enhancement relies on the unique optical and plasmonic properties associated with metallic nanostructures. Conventionally, plasmonic nanostructures are often designed to offer intense localized surface plasmon resonance (LSPR) caused by the collective oscillation of electrons when excited by light of a resonant frequency. Areas of high electric field found in the interstitial gaps between metal surfaces are often referred to as SERS hot-spots.^1,2,9^ The SERS signal generated from molecules that get trapped in the hot spots is significantly amplified. Hot spots can also be generated at the tips or edges of a noble metal surface.^1,2,9^ Ideal SERS-active nanostructures for sensing applications exploit this phenomenon by drastically enhancing Raman scattering in a reproducible and uniform manner.^3^ However, many of the tuneable SERS-active nanostructures involve complex synthetic processes and suffer from poor structural reproducibility often resulting in widely distributed enhancement factors (EFs) limiting clinical application by hindering the ability to quantify biomarkers in a reliable manner.^2,7,9,10^

As mentioned previously, reliability and robustness have been among the key challenges for SERS measurements especially under aqueous conditions. A number of approaches have been employed by researchers to create high performance SERS-active NPs,^11–14^ including the variation of physical properties associated with the particles such as size,^15–17^ shape/morphology,^18–20^ material composition,^21–23^ and gap distance/interstitial junction.^24–26^ Specific examples include work by Yang *et al.* that introduces the synthesis of bimetallic Ag@Au nanocubes by deposition of Au atoms on the surface of the Ag nanocubes.^27^ A similar study by Gopalakrishnan *et al.* demonstrates the synthesis of bimetallic 3-D nanostar dimers in a ring cavity to further enhance the overall SERS performance.^28^ In both studies, SERS-active bimetallic NPs with unique morphologies have been demonstrated to exhibit greater SERS enhancement of associated analytes when compared to bare unmodified NPs.^29^ However, although these SERS-active NPs offer large EFs, the poor reproducibility and instability resulting from the desorption of Raman reporters through competitive binding of other molecules cannot be ignored. Additionally, many SERS-active NPs require further subsequent surface functionalization to enhance stability. To be applied in a real-world application, integration of these SERS-active NPs in diagnostic assays requires further addition of targeting moieties to adhere to the surface such as aptamers,^30,31^ antibodies,^32–34^ oligonucleotide probes^35,36^ and Raman reporter molecules (RRMs).^37^ This mixed monolayer often results in competitive binding on the exposed NP surface, causing inequivalent surface deposition. Loss of RRMs due to surface desorption limits the SERS sensitivity once applied to a diagnostic assay.

To overcome these challenges, we have devised a synthesis method for the development of novel core–satellite nanostructures whereby a gap is created between the core and the satellite using a heterobifunctional PEG linker. The effect of changing the length of the PEG linker on the overall SERS performance was explored through assessing the two most important key parameters of signal intensity and reproducibility. To evaluate the SERS performance, a commonly employed RRM, malachite green isothiocyanate (MGITC), was utilized. In particular, a core–satellite NP assembly was chosen due to hot-spot generation through core–satellite plasmonic coupling and satellite–satellite plasmonic coupling. This synergistic combination leads to a strong SERS enhancement due to the high electromagnetic coupling between the two moieties.^38^ Based on a theoretical prediction by Singamaneni *et al.*, it was illustrated that the electric field intensity generated at the interstitial space between the core and satellite NPs is about 660× higher than that at the surfaces of the cores. Therefore, in comparison to conventionally aggregated NP systems, core–satellite assemblies have better repeatability due to the high affinity for core–satellite interaction.^39^ To synthesize these nanoparticle systems, bottom-up approaches are predominantly utilized using either organic^40^ or inorganic templates^41^ as core nanostructures, while smaller inorganic nanoparticles such as gold, silver, or semiconductor NPs are explored as satellites. The nanospacer linkers that are commonly employed include (1) protein-based assemblies,^42^ (2) polymer-based assemblies,^43–45^ (3) DNA-based assemblies,^37,46–50^ and (4) molecular linkers that directly bridge the plasmonic units using either covalent or electrostatic interactions of the functional groups.^38,51–53^ For example, Chen *et al.* demonstrated the synthesis of a streptavidin-coated polymeric bead core–Au satellite assembly *via* DNA linkers.^54^ Two different types of DNA linker were immobilized on the surface of the nanoparticles to enhance stability, and a pre-treatment step for both linkers and gold nanoparticles (AuNPs) was required to initiate the functionalization. However, the use of DNA is often laborious and expensive. Typically, DNA mediated core–satellite assemblies require at least ∼17 hours to complete the reaction with many subsequent centrifugation steps.^37,46–50^ Moderate enhancing capabilities are observed within the DNA mediated assembly due to the intrinsic DNA length required to ensure stable hybridization precluding tunability of the interparticle gap distances between the core and satellite nanostructures.^54,55^ Similar limitations are seen utilizing bulky macromolecules such as protein-mediated assembly or branched polymer-mediated assembly. On the other hand, efficient distribution of molecular linkers on the nanoparticle surface is hampered due to the irreversible colloidal instability they introduce to the NP system.^56^ To overcome this challenge, there is a need to develop a more cost-effective and scalable synthesis of these CSNPs with tunable SERS hotspot generation.

In this study, we report a facile synthesis method of gold core–satellite nanoparticles that exhibit high SERS intensity with great reproducibility. We explore these SERS hotspot generations by immobilizing linear heterobifunctional polyethylene glycol (PEG) that is composed of an amine and a thiol group. This linear PEG chain serves as a tunable nanospacer for the core–satellite structure. By altering the distance between the core and satellite nanostructures, we can induce controllable SERS hotspot generation. The SERS hotspot generation can be tuned by altering the distance between the nanostructures by using various PEG polymer thicknesses. The core–satellite nanoparticles were generated through immobilization of the satellite nanoparticles through electrostatic interactions of the positively charged amine groups of the PEG chain and the negatively charged citrate stabilized seed nanoparticles. It is shown that these core–satellite gold nanoparticles are synthesized in an easy and low-cost manner and generate strong and reproducible SERS signals, thus addressing the need for a SERS-active nanostructure with high sensitivity and stability.

## Materials and methods

### Chemicals

Gold(iii) chloride trihydrate (HAuCl_4_·3H_2_O, >99.9%), trisodium citrate dihydrate (≥99.0%), and malachite green isothiocyanate (MGITC) were all purchased from Sigma-Aldrich. Linear heterobifunctional polyethylene glycol (SH-PEG-NH_2_) linkers with a molecular weight (MW) of 1000 Da were obtained from Nanosoft Polymers (North Carolina, USA), and linkers weighing 2000 Da and 3500 Da were obtained from JenKem Technology (Texas, USA). Ultrapure water (Milli-Q) was used for the preparation of all solutions.

### Methods

#### Synthesis of 30 nm spherical core AuNPs

30 nm AuNPs were synthesized *via* the Turkevich method.^57^ 10 mL of 5 mM auric chloride (HAuCl_4_) was initially added to 85 mL of distilled water, while vigorously stirring and heating. 4.5 mL of 0.03 M trisodium citrate dihydrate was added immediately to the gold solution once the solution started to boil. The initial light-yellow solution gradually turned dark wine red, indicating successful formation of AuNPs. Throughout this manuscript, we will refer to SH-PEG_1000_-NH_2_ as PEG_1000_, SH-PEG_2000_-NH_2_ as PEG_2000_, and SH-PEG_3000_-NH_2_ as PEG_3000_. Additionally, we will refer to gold core–satellite nanoparticles (Au-CSNPs) as CSNPs.

#### Synthesis of 18 nm satellite AuNPs

A similar method was used to synthesize the 18 nm satellite AuNPs. In a 100 mL glass flask, 10 mL of 5 mM HAuCl_4_ was added to 85 mL of distilled water under magnetic stirring. The solution was allowed to boil and 5 mL of the 0.03 M trisodium citrate dihydrate was immediately added to the solution. Similarly, the initial light-yellow solution turned light wine red in color, forming the satellite AuNPs.

#### Synthesis of core–satellite NPs

1 mL of synthesized 30 nm AuNPs was centrifuged at 7000 rpm for 8 min. The pellet was then used for SH-PEG_*x*_-NH_2_ functionalization (where *x* is 1000, 2000 or 3500 and refers to PEG with a MW of 1000, 2000, or 3500 Da) and RRM functionalization. The RRM (MGITC) was attached to the AuNPs by exploiting the favourable Au–isothiocyanate interaction. After initial centrifugation prior to the surface functionalization, the absorbance (optical density, OD) of each nanoparticle solution was 0.54 A.U. at *λ*_max_ = 525 nm when 1 mL of DI water was added to resuspend the pellet with a calculated concentration of 0.16 nM.

#### RRM & linear bifunctional thiol-PEG_*x*_-amine functionalization

100 μL of 0.05 mg mL^−1^ SH-PEG_1000_-NH_2_ (PEG_1000_) was added to the pellet along with 150 μL of DI water, and 75 μL of 10^−5^ M MGITC while stirring. Assuming a total volume of 325 μL, the PEG_1000_ concentration in the solution is 15.3 μM. The solution was then allowed to incubate for 30 min and centrifuged at 7000 rpm for 8 min to remove unbound PEG_1000_ and MGITC. 250 μL of DI water was then added followed by another 75 μL of 10 μM MGITC to ensure optimal saturation of RRMs on the NP surface. The solution was allowed to incubate for another 30 min and centrifuged at 7000 rpm at 8 min to remove excess MGITC. The sample was then washed by adding 1 mL of DI water to the pellet followed by subsequent centrifugation. The final pellet was then resuspended in 1 mL of DI water. 100 μL of the sample was then utilized to characterize optical stability with a microplate reader and 100 μL of the sample was utilized to characterize SERS performance as a control. Similarly, 150 μL of 0.05 mg mL^−1^ SH-PEG_2000/3500_-NH_2_ (PEG_2000_ & PEG_3500_) was added to the pellet along with 150 μL of DI water, and 75 μL of 10 μM MGITC which was added rapidly while stirring. The solution was then allowed to incubate for 30 min and centrifuged once again at 7000 rpm for 8 min to remove excess PEG_2000/3500_ and MGITC. 250 μL of DI water was then added followed by another 75 μL of 10 μM MGITC. The solution was allowed to incubate for another 30 min and centrifuged at 7000 rpm for 8 min to remove excess MGITC. The sample was then washed by adding 1 mL of DI water to the pellet followed by a subsequent centrifugation. The final pellet was then resuspended in 1 mL of DI water. 100 μL of the sample was then utilized to characterize optical stability with the microplate reader and 100 μL of the sample was utilized to characterize SERS performance as a control.

#### Attachment of 18 nm satellite NPs

For the core–satellite NPs modified with PEG_1000_, 800 μL of the PEGylated AuNPs (OD: 0.435 ± 0.0077 A.U., *λ* = 530 nm) were then added dropwise under magnetic stirring to 200 μL (OD 0.727 A.U., *λ* = 520 nm) of the 18 nm satellite AuNPs. Similarly, for PEG_2000_ and PEG_3500_ functionalization, 800 μL of the PEGylated AuNPs (OD: 0.435 ± 0.0077 A.U., *λ* = 530 nm) were then added dropwise under magnetic stirring to 250 μL (OD 0.727 A.U., *λ* = 520 nm) of the 18 nm satellite AuNPs. PEGylated AuNPs were added dropwise until the solution turned from the initial light pink to bluish in color forming the distinct core–satellite AuNPs instantaneously. The assembly of the CSNPs took 2–3 hours and was repeated 4 times for statistical analysis.

#### Characterization of the core–satellite NPs

A Tecan Infinite 200 Pro microplate reader was utilized to characterize the optical stability of the core and satellite AuNPs which demonstrated strong extinctions at 525 nm and 520 nm, respectively. Surface charge measurements at each functionalization step were then carried out utilizing a Litesizer1000 to confirm stable formation and functionalization of the CSNPs. TEM images were taken using a JEOL 1200 and image analysis was performed in ImageJ to evaluate the size of the CSNPs. For transmission electron microscopy, 8 μL of the prepared AuNPs was dropped onto carbon film-coated copper grids and allowed to dry at room temperature.

#### Sample preparation and SERS measurements

SERS measurements were performed using a benchtop Raman device with an excitation laser of 785 nm and collected at 1 s integration time. Samples were fixed at a concentration of 0.19 nM (OD of 0.4 A.U.). All spectra were baseline corrected.

## Results and discussion

### Synthesis of the gold core–satellite NPs

**Fig. 1:**
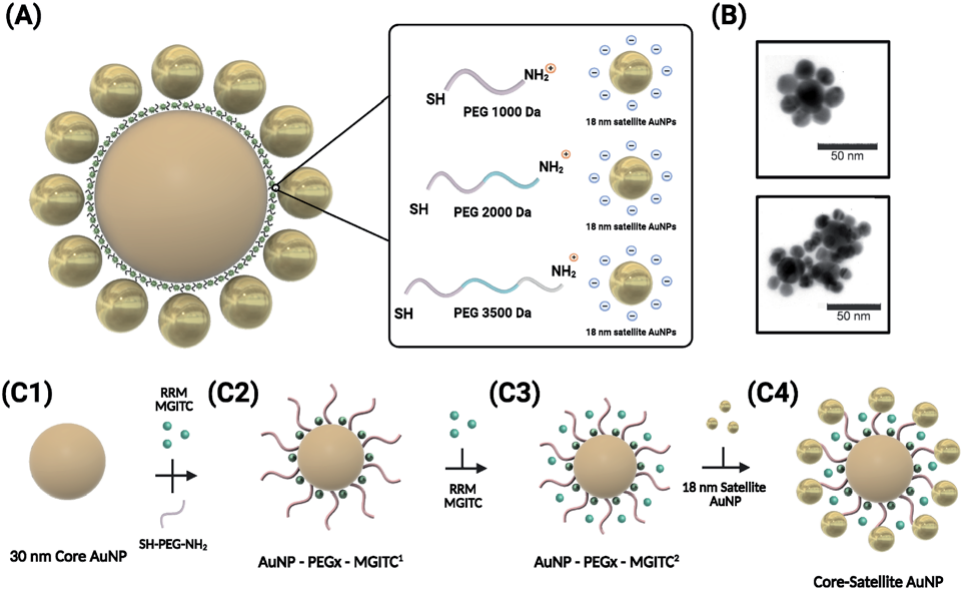
Schematic illustration of the synthesis of the core–satellite AuNPs. (A) The overall morphology of the CSNPs with MGITC entrapped between the core and satellite nanostructures. A tunable hot-spot formation can be achieved by modulating PEG lengths. (B) TEM images of CSNPs–PEG_1000_–MGITC^2^ forming a discrete core–satellite nanostructure. (C) Illustration of the nanoparticle assembly forming the distinct core–satellite NPs through functionalization of heterobifunctional PEG and MGITC.

Fig. 1 depicts the synthesis and assembly process of the CSNPs. The core and satellite AuNPs were synthesized *via* the Turkevich method^57^ (verified by TEM in Fig. 3A). In this assembly, highly stabilized CSNPs were synthesized by anchoring linear heterobifunctional polyethylene glycol (SH-PEG_*x*_-NH_2_, where *x* denotes the various MWs of PEG (1000, 2000, and 3500)) on the surface of the core AuNPs. The SH-PEG_*x*_-NH_2_ (referred to as PEG_*x*_ in this paper) serves as a tunable nanospacer altering the distance between the core and surface immobilized satellite nanoparticles. Additionally, Raman reporter molecules (MGITC) were simultaneously anchored on the surface of the core particles. Here, competition for surface attachment on the AuNP core occurs between PEG_*x*_ and MGITC to form a mixed monolayer. Due to the comparatively weak isothiocyanate binding of the MGITC, PEG_*x*_ adsorbs more readily allowing more active groups for binding of the satellite NPs. The use of the PEG_*x*_ linker allows MGITC to become trapped at the interface of the core and satellite particles, which is an area of high electric field density amenable to large increases in SERS enhancement. Exploration of different PEG lengths offers a facile, cost-effective method to tune the distance between the core and satellite nanostructures, thereby modulating the SERS signal through hotspot generation.

**Fig. 2:**
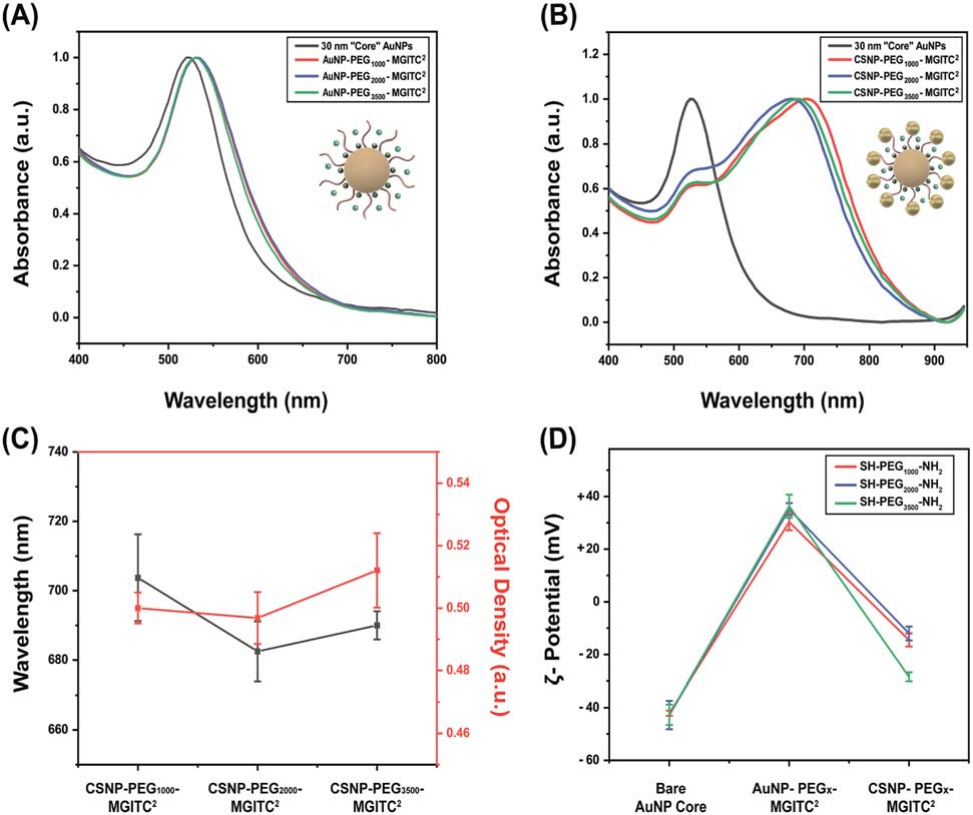
(A) UV-Vis spectra to verify the optical stability of the 30 nm AuNP core nanoparticles and PEG_*x*_–MGITC^2^ modified core nanoparticles at various polymer thicknesses (MW: 1000 Da, 2000 Da, and 3500 Da), (B) UV-Vis spectra illustrating the spectral shift after formation of CSNPs modified by PEG with different polymer thicknesses, (C) CSNP wavelength shift for each PEG polymer thickness and optical density after synthesis, and (D) *ζ*-potential measurements illustrating successful surface modification at each functionalization step through surface charge differences for each synthesized CSNP.

**Fig. 3:**
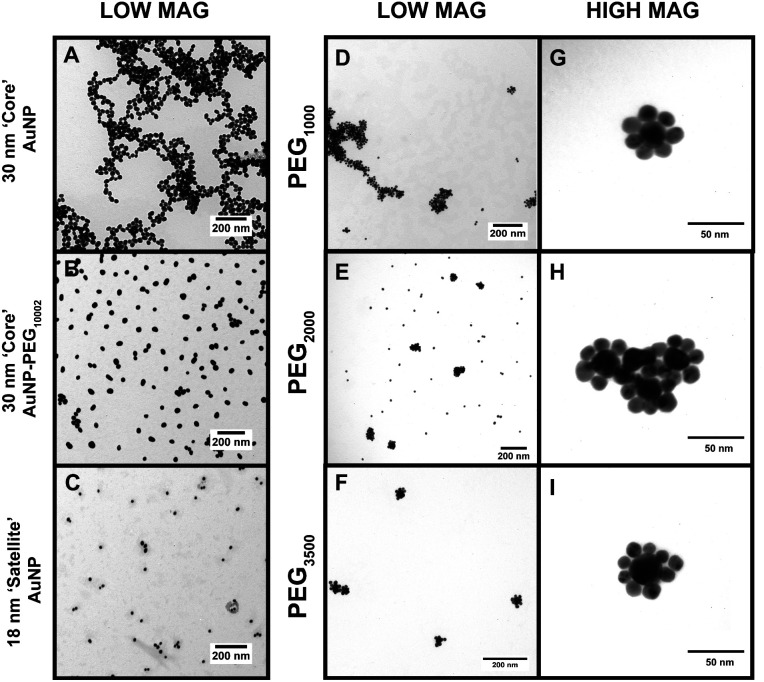
TEM images of the CSNP assemblies with (A) 30 nm core AuNPs at low magnification, (B) 30 nm core AuNP–PEG_1000_–MGITC^2^, (C) 18 nm satellite AuNPs, (D–F) CSNP–PEG_*x*_–MGITC^2^ with MWs of 1000 Da, 2000 Da, and 3500 Da, respectively, all at low magnification, and (G–I) CSNP–PEG_*x*_–MGITC^2^ with MWs of 1000 Da, 2000 Da, and 3500 Da, respectively, at high magnification.

The simultaneous attachment of PEG_*x*_ and MGITC results in modified AuNP–PEG_*x*_–MGITC^1^ as shown in Fig. 1(C2), where MGITC^1^ denotes one loading cycle of MGITC. An additional loading of MGITC after PEG_*x*_ modification was made to ensure optimal RRM entrapment between the core and satellite nanostructures, which results in AuNP–PEG_*x*_–MGITC^2^ as shown in Fig. 1(C3). Following this reaction, 18 nm AuNP seeds were then deposited onto the AuNP–PEG_*x*_–MGITC^2^ as shown in Fig. 1(C4). CSNP formation and morphology were characterized using UV-Vis spectrophotometry (UV-Vis), *ζ*-potential measurements, and transmission electron microscopy (TEM).

### Characterization of the CSNP assembly

UV-Vis measurements obtained for the 30 nm core AuNPs exhibited a LSPR peak at 525 nm shown in Fig. 2A. Successful functionalization of the core AuNPs with the PEG_*x*_–MGITC^2^ was confirmed upon observation of a 5 nm redshift to 530 nm caused by a change in the refractive index. Experimental observations revealed that the addition of too much MGITC onto the AuNP surface in one go would lead to immediate particle instability due to a reduction in charge repulsion between particles causing AuNP aggregation. Therefore, PEG_*x*_ and MGITC were added simultaneously to enhance the stability of the nanoparticles as well as to serve as an anchor site for satellite AuNP attachment. Due to the simultaneous attachment of MGITC and PEG_*x*_, competitive binding between the two moieties arises. ESI Fig. S1(A–C)† report the hydrodynamic size of 36.67 nm and SERS enhancement of CSNPs–PEG_1000_–MGITC^1^ and CSNPs–PEG_1000_–MGITC^2^. Fig. S1(B and C)† illustrate minimal enhancement when MGITC was only deposited once due to the competitive binding between the two moieties.

To control the simultaneous loading of MGITC and PEG_*x*_, various amounts of PEG_*x*_ deposited on the surface of the nanoparticles were explored. The deposition of MGITC and different PEG lengths were modulated by fixing one parameter. In this case, MGITC was fixed at a concentration of 10^−5^ M and different concentration ranges of PEG_1000_ were explored ranging from 1.53 mM (0.5 mg PEG) to 153 μM (0.05 mg PEG) and 15.3 μM (0.005 mg PEG) attached to a fixed concentration of AuNPs (OD of 0.6 A.U.) shown in Fig. S2.† Fig S2A† illustrates the UV-Vis measurement for the 30 nm AuNP core exhibiting a LSPR peak at 525 nm, and a shift to 530 nm confirmed the successful attachment of PEG_1000_ at all concentration ranges. Through *ζ*-potential measurements, a minimum threshold amount of PEG_*x*_ was identified for each MW of PEG_*x*_. Fig. S2B† reports the minimum saturation point for PEG_1000_ attachment at a fixed 10^−5^ M concentration of MGITC, which is 15.3 μM PEG_1000_. SERS measurements were also conducted to evaluate whether enhancement of MGITC^1^ and various concentration ranges of PEG_1000_ are observed shown in Fig. S2(C and D).† The simultaneous addition of MGITC and PEG_*x*_ leads to competitive binding of the two moieties on the surface of the nanoparticle leading to minimal SERS enhancement due to poor attachment of MGITC. As mentioned previously, an additional MGITC loading was added to the surface after PEG_*x*_ functionalization to ensure effective deposition of MGITC in the nanoparticle assembly.

Attachment of the satellite nanoparticles to the CSNPs was achieved by adding a fixed concentration of 18 nm satellite nanoparticles to the solution containing PEG_*x*_ modified core AuNPs. In this experiment, a volumetric ratio of 1 : 4 Au core–Au satellite seed was used for CSNPs–PEG_1000_–MGITC^2^. Meanwhile, a volume ratio of 1 : 3 Au core–Au satellite seed ratio was used for both CSNPs–PEG_2000_–MGITC^2^ and CSNPs–PEG_3500_–MGITC^2^. It was noticed that if we increased the ratiometric amount of Au satellites above 3 the particles became destabilized causing sedimentation. This may be attributed to the reduction of coulombic repulsion between assemblies due to excess satellite NP inclusion in the synthesis step. The initial wine-red color of the core turns light blue due to plasmonic coupling between the core and satellite particles which indicates successful gold satellite anchoring. UV-Vis was used to monitor the color change (Fig. 2B and C), and a red shift from the *λ*_ma*x*_ at 530 nm to higher wavelengths of 704 nm ± 12.5 nm, 683 ± 8.7 nm, and 690 ± 4.08 nm was observed for CSNPs modified with PEG_*x*_–MGITC^2^ with MWs of 1000 Da, 2000 Da, and 3500 Da, respectively. The change in extinction profile morphology is observed after the addition of the 18 nm satellite AuNPs and is believed to be caused by the newly created plasmon coupling between the tightly packed core and satellite nanostructures. *ζ*-Potential measurements further confirmed the successful assembly of CSNPs by assessing the change of surface charge on the nanoparticle surface illustrated in Fig. 2D. The bare AuNP displayed an average *ζ*-potential of −42.1 ± 0.38 mV. During the assembly of the core–satellite nanoparticles, the *ζ*-potential increased to +30.29 ± 1.04 mV, +35.16 ± 0.78 mV, and +36.3 ± 1.48 mV after the addition of the PEG_*x*_–MGITC with MWs of 1000 Da, 2000 Da, and 3500 Da, respectively. Due to the heterobifunctional nature of the PEG_*x*_ chain, the thiol groups (SH) favourably bind to the AuNP surface through Au–S interactions while the amine (NH_2_) groups are left exposed on the surface resulting in an overall positive charge on the nanoparticle surface. Previous studies have reported that the gold–sulphur (Au–S) covalent interaction is exceptionally strong. Meanwhile, amine groups have been shown to bind through electrostatic interaction with the negatively charged AuNP surface.^58,59^ Specifically, it has been noted that thiol groups have shown the strongest binding affinity towards the surface of noble gold nanoparticles due to the nature of the covalent interaction or “chemisorption binding”.^59,60^ In comparison, amine groups are noted to have weak covalent bonding with the negatively charged surface of the gold nanoparticles.^58,59,61^ Specifically, a study reported by Ftouni *et al.* evaluated the effects of citrate capped AuNP binding on two linkers, (3-aminopropyl)triethoxysilane (APTES) and (3-mercaptopropyl)triethoxysilane (MPTES).^61^ MPTES was utilized for its thiol functionality, and APTES linker was chosen due to the weak covalent affinity of the amino group to the gold nanoparticles reported previously as well by Genzer *et al.*^61,62^ In this work, they evaluated the catalytic activity and utilized scanning electron microscopy and electron probe microanalysis to confirm the presence of the gold nanoparticles.^61^ The author reported that using MPTES linkers with thiol modification exhibited better stability due to stronger bonding to the gold nanoparticles.^61^ Additionally, a study by Tao *et al.* evaluated the effects of anchoring groups such as amine, thiol, and carboxylic acid functional groups on the single-molecule conductance with gold.^59^ Binding strength information was obtained by measuring the average length over which one can stretch each molecular junction until it breaks, which varies in the order of Au–S > Au–NH_2_ > Au–COOH, which is consistent with the binding strengths of the three anchoring groups to gold.^59^ Additionally, a study by Cortie *et al.* confirmed the weak adsorption of amine compounds with only significant binding in the under-coordinated atom site of the gold structure.^60^ The authors confirmed these findings using a density functional theory study of the adsorption energetics of various amine compounds on the gold surface.^60^ In our work, it is believed that preferential binding of the thiol groups occurs on the NP surface due to the resulting overall positive *ζ*-potential measurement after functionalization with each respective PEG length chain. The surface charge switch from a negatively charged nanoparticle surface to a positively charged surface further confirmed the successful functionalization of the PEG_*x*_–MGITC. The amine groups serve as active binding sites for satellite particle attachment *via* AuNP–amine electrostatic interaction.^63^ Through this electrostatic interaction between the amine active groups and negatively charged citrate stabilized surface of the satellite particles, the well-defined CSNPs display a mean *ζ*-potential of −14.50 ± 0.83 mV, −12.0 ± 0.878 mV, and −28.5 ± 0.56 mV, respectively, for PEG_*x*_–MGITC with MWs of 1000 Da, 2000 Da, and 3500 Da.

### Examining the morphological structure of the CSNP assembly

The TEM images in Fig. 3 offer a qualitative image quantification of each characterization step of the CSNP assembly at different MWs. Herein, TEM-based quantification of core sizes was measured to evaluate morphological changes after each functionalization step. Fig. 3A shows a TEM image of discrete unmodified core AuNPs with an average diameter of 30.07 ± 6.68 nm. Fig. 3B illustrates a TEM image captured for core AuNPs modified with PEG_1000_–MGITC^2^, and Fig. 3C shows the satellite AuNPs with an average diameter of 18.39 ± 3.719 nm. Fig. 3D–F report low magnification images of the synthesized CSNPs with PEG_*x*_ of various MWs (1000 Da, 2000 Da, and 3500 Da), respectively. It is common that the nanoparticles tend to cluster together as part of the drying process, as the solvent evaporates.^64,65^ Due to the decrease in volume as the solvent evaporates, electrostatic imbalances occur between the nanoparticles causing them to cluster together.^64,65^Fig. 3G–I show high magnification images for each of the CSNPs synthesized. Fig. 3G–I illustrate the clear attachment of the 18 nm AuNP satellite seed on the 30 nm core AuNP surface forming a satellite-like morphology for all MWs of PEG_*x*_–MGITC^2^. Through ImageJ analysis, the number of satellites detected around each nanoparticle core was 6 ± 1, 8 ± 2, and 8 ± 2, respectively, for PEG_*x*_ (MW: 1000 Da, 2000 Da, and 3500 Da). ESI Fig. S3† further shows all high magnification images captured for CSNPs synthesized with different PEG length chains. These TEM images illustrate the assembly process which demonstrates the attachment of the 18 nm Au satellite seeds forming discrete core–satellite nanostructures. Herein, controlling the amount of PEG_*x*_ and MGITC is essential as an ideal amount of NH_2_ active sites are required for proper ∼18 nm satellite attachment while also ensuring that MGITC is entrapped on the nanoparticle surface.

### Investigating SERS enhancement as a factor of PEG length

**Fig. 4:**
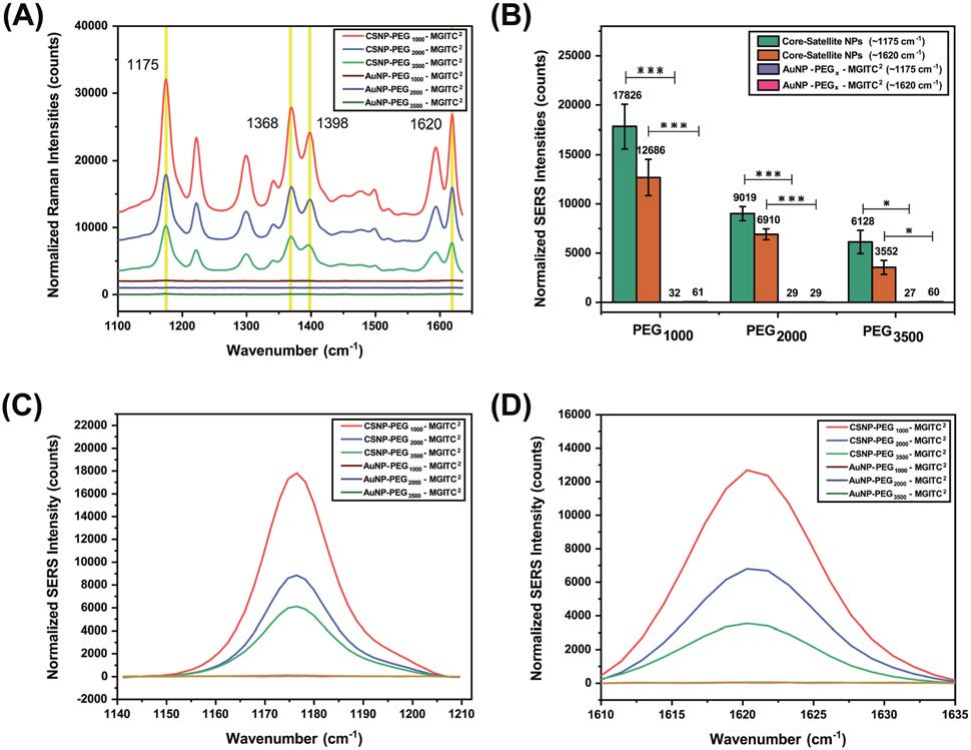
(A) The SERS spectra generated from MGITC adsorbed onto the surface of CSNPs functionalized with PEG_*x*_ (MW: 1000 Da, 2000 Da, and 3500 Da) and AuNP cores modified with PEG_*x*_ (MW: 1000 Da, 2000 Da, and 3500 Da). (B) The difference in SERS peak intensity observed for the CSNPs and respective AuNP ‘core’ controls of characteristic MGITC peaks positioned at ∼1175 cm^−1^ and ∼1620 cm^−1^. (C and D) SERS spectra of individual MGITC peaks (1175 cm^−1^ (C) and 1620 cm^−1^ (D)) for all CSNPs and respective AuNP controls. Notice that without attachment of the satellites hardly any SERS peak is observed.

The overall goal of this study was to evaluate the effects of the PEG_*x*_-polymer length between the core and satellite nanostructures on overall SERS performance. Previous studies have shown that core size had only a minor effect on the overall SERS enhancement and that the majority of SERS originated from satellite–satellite interactions.^66^ Therefore, to observe the enhancements based on PEG lengths, a fixed concentration and volume of satellite nanoparticles were deposited onto the heterobifunctional linkers as various MWs of PEG have different surface coverage densities and exposed NH_2_ active sites. Additionally, equimolar concentrations of CSNPs–PEG_*x*_–MGITC^2^ and AuNP–PEG_*x*_–MGITC^2^ were analysed at an excitation of 785 nm for SERS analysis. As seen in Fig. 4, the AuNP–PEG_*x*_–MGITC^2^ exhibits limited SERS enhancement despite the loading of the MGITC. As seen in ESI Fig. 2C,† the addition of the linear bifunctional PEG interfered with the SERS signal enhancement of the RRM. However, after the addition of the satellite nanoparticles, an intense signal amplification of the RRM was observed as shown in Fig. 4A–D. MGITC Raman spectral bands were observed and tentatively assigned at ∼1170 cm^−1^ (corresponding to the in-plane aromatic C–H bending vibration), ∼1368 cm^−1^ (N–C stretching), ∼1398 cm^−1^ (C–C and C–H in-plane motion), and ∼1620 cm^−1^ (N–C bond and C–C stretching). For further studies, we evaluated the characteristic intensities of MGITC peaks at ∼1175 cm^−1^ and ∼1620 cm^−1^ post baseline correction. CSNPs modified with PEG_1000_–MGITC^2^ that were excited at 785 nm realized the highest signals: 17 826 and 12 686 for 1175 cm^−1^ and 1620 cm^−1^, respectively. There is a clear decrease in SERS intensity for CSNPs modified with PEG_2000_–MGITC^2^ and CSNPs modified with PEG_3500_–MGITC^2^ with reported SERS intensities of around 8772 and 6128 at ∼1175 cm^−1^ as well as 6765 and 3552 at ∼1620 cm^−1^, respectively. Herein, it is reported that SERS enhancement increased in the order of CSNPs modified with PEG_1000_ > PEG_2000_ > PEG_3500_. This is most likely due to the formation of smaller nanogaps when using linkers containing PEG_1000_ compared to the larger gaps created when using PEG_3500_. The smaller gap of the core–satellite interface is ideal for plasmon coupling/hot-spot formation to occur, resulting in a larger SERS signal being generated from trapped MGITC RRMs. In addition, it is important to keep in mind that the satellite nanoparticle size is crucial in obtaining appropriate SERS enhancement. As shown in ESI Fig. S4,† we report the use of 2 nm satellite AuNPs in the same nanoparticle assembly where minimal SERS enhancement was observed when 2 nm satellite AuNPs are used.

### Long-term optical stability of the CSNP assembly

**Fig. 5:**
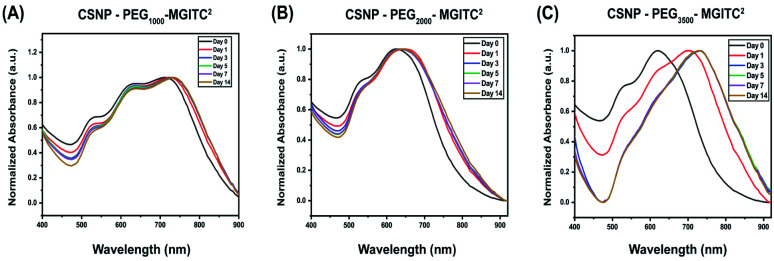
(A–C) Optical stability of CSNPs synthesized with various polymer thicknesses at 0, 1, 3, 5, 7, and 14 days. (A) CSNPs with PEG_1000_–MGITC^2^ at a volume ratio of 1 : 4 Au satellite to Au core NPs, (B) CSNPs with PEG_2000_–MGITC^2^ and (C) CSNPs with PEG_3500_–MGITC^2^ at a volume ratio of 1 : 3 Au satellite to Au core NPs.

To characterize the long-term stability of CSNP nanostructures in an aqueous solution sensing application, UV-Vis stability measurements were obtained at various time points ranging from 0 to 14 days after synthesis. Fig. 5A and ESI Fig. S5† report a slight blue shift in wavelength, and a decrease in optical density is observed for CSNPs synthesized from the bifunctional linkers containing different PEG lengths.

Specifically, Fig. 5A demonstrates a blue shift of CSNPs–PEG_1000_–MGITC^2^ from an average of 725 to 730 nm in the span of 14 days. Additionally, a calculated 19.48% loss of particles was observed after 14 days based on the decrease in absorbance (optical density) illustrated in ESI Fig. S5A.† In both CSNPs modified with PEGs with MWs of 2000 Da and 3500 Da, a spectral red shift was observed. CSNPs–PEG_2000_–MGITC^2^ exhibited a spectral red shift from 625 nm to 640 nm after 14 days, and the loss of NPs based on the decrease of absorbance was calculated to be around 30.24% after 14 days as shown in Fig. 5B and ESI Fig. S5B.† CSNPs–PEG_3500_–MGITC^2^ reported the largest spectral red shift from 620 nm to 730 nm stabilizing after 5 days. Similarly, CSNPs–PEG_3500_–MGITC^2^ reported a 47.68% decrease in absorbance after 14 days. Herein, it was observed that CSNPs–PEG_1000_–MGITC^2^ < CSNPs–PEG_2000_–MGITC^2^ < CSNPs–PEG3500–MGITC^2^ is the order of lowest to greatest loss of particles after 14 days.

### Measuring the SERS signal generated from the CSNPs for two weeks

**Fig. 6:**
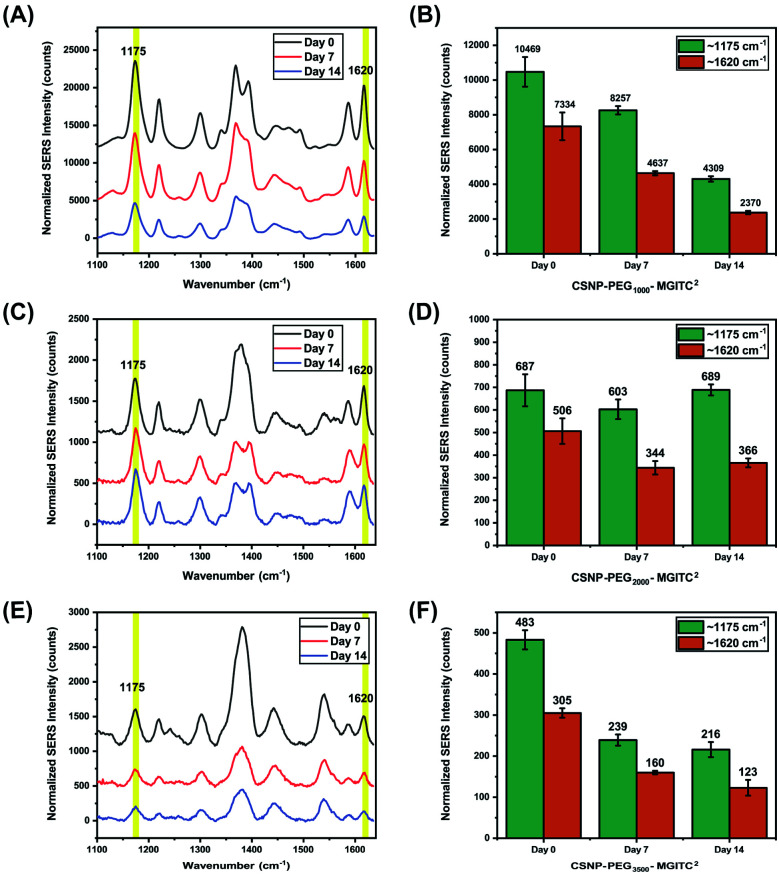
SERS measurements conducted at 0, 7, and 14 days for CSNPs with various gap distances: SERS retention of (A and B) CSNPs with PEG_1000_–MGITC^2^, (C and D) CSNPs with PEG_2000_–MGITC^2^ and (E and F) CSNPs with PEG_3500_–MGITC^2^, confirming the distinct MGITC characteristic peaks at 1175 cm^−1^ and 1620 cm^−1^.

The long-term SERS activity of CSNPs composed of different PEG lengths in aqueous solution was characterized by taking SERS measurement of the CSNPs at 0, 7, and 14 days shown in Fig. 6. The CSNPs–PEG_*x*_–MGITC^2^ were characterized with a laser excitation of 785 nm at 1000 ms integration time. Based on the results, a retention of 41.16% of the SERS signal after 14 days is observed for CSNPs synthesized with PEG_1000_–MGITC^2^ at ∼1170 cm^−1^ corresponding to the in-plane aromatic C–H bending vibration. Additionally, a similar trend is observed for ∼1620 cm^−1^ corresponding to the N–C (ϕ bond) and C–C stretching with a signal retention of 32.3% after Day 14. CSNPs–PEG_3500_–MGITC^2^ exhibited a similar trend after 14 days with a SERS signal retention of around 44.7% and 40.32% at ∼1170 cm^−1^ and 1620 cm^−1^, respectively. Meanwhile, CSNPs–PEG_2000_–MGITC^2^ exhibited a retention of around 100.2% and 72.3% at ∼1170 cm^−1^ and 1620 cm^−1^. This could be attributed to the aggregation of the particles on Day 14 which could possibly cause a slight increase of signal.

## Conclusions

In this work, CSNPs modified with PEG with various MWs (1000 Da, 2000 Da, and 3500 Da) were successfully achieved using heterobifunctional PEG containing thiol (SH) and amine (NH_2_) functional groups. Successful formation of CSNPs was verified through UV-Vis measurements, confirming successful functionalization through spectral shift, and *ζ*-potential measurements by quantitatively measuring the change in surface charge. Successful modulation of PEG_*x*_ and MGITC was achieved resulting in discrete CSNP formation. TEM images further confirmed the morphological changes. A tunable SERS hot spot was generated through the exploration of PEG with different length chains. Herein, it was observed that CSNPs–PEG_1000_–MGITC^2^ offered the highest signal enhancement when compared to longer PEG_*x*_ polymer chains. Herein, it was observed that CSNPs–PEG_1000_–MGITC^2^, CSNPs–PEG_2000_–MGITC^2^, and CSNPs–PEG_3500_–MGITC^2^ have the lowest to highest loss of particles after 14 days as well as having the highest to lowest SERS signal enhancement due to nanogap distances. As for SERS signal retention, it was observed that CSNPs–PEG_2000_–MGITC^2^ retained most of the SERS signal at 14 days as opposed to the other PEG length chains. This nanoparticle system has a free surface monolayer that can further stabilize the NP system while also allowing application in a diagnostic assay by functionalization with target moieties such as antibodies, aptamers, and DNA oligonucleotide probes.

## Conflicts of interest

There are no conflicts to declare.

## Supplementary Material

NA-004-D1NA00676B-s001
