# Supplementary material for: Synthesis of SERS-active core–satellite nanoparticles using heterobifunctional PEG linkers
Source: Nanoscale Adv. 2021 Nov 15;4(1):258–67. doi: 10.1039/d1na00676b (PMC9417690; doi:10.1039/d1na00676b)
Supplement: NA-004-D1NA00676B-s001 [file NA-004-D1NA00676B-s001.pdf]

Electronic Supplementary Material (ESI) for Nanoscale Advances.
This journal is © The Royal Society of Chemistry 2021

SUPPLEMENTARY FIGURES

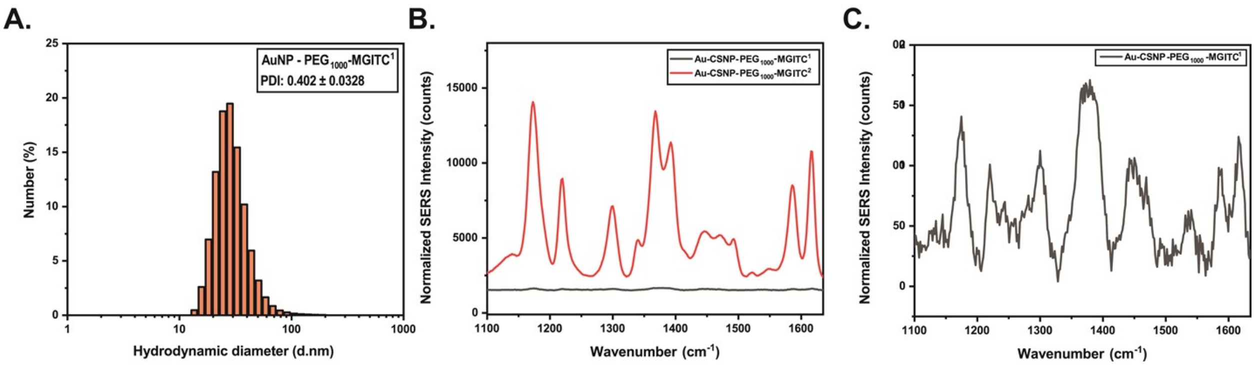

Supplemental Figure S1A. Reports hydrodynamic diameter size distribution of Core AuNPs modified with SH-PEG_{1000}-MGITC[1] with an average diameter 36.67 nm with a PDI of 0.402 ± 0.0328. **Fig S1B.** Illustrates SERS intensity measurement comparison between Au- Core satellite NPs synthesized with 1 loading of MGITC and 2 loading of MGITC with MGITC characteristic peaks evident at ~1170 cm^{-1} and ~1620 cm^{-1}. **Fig S1C.** Reports graphical illustration of CSNPs with 1 loading of MGITC illustrating MGITC characteristic peaks.

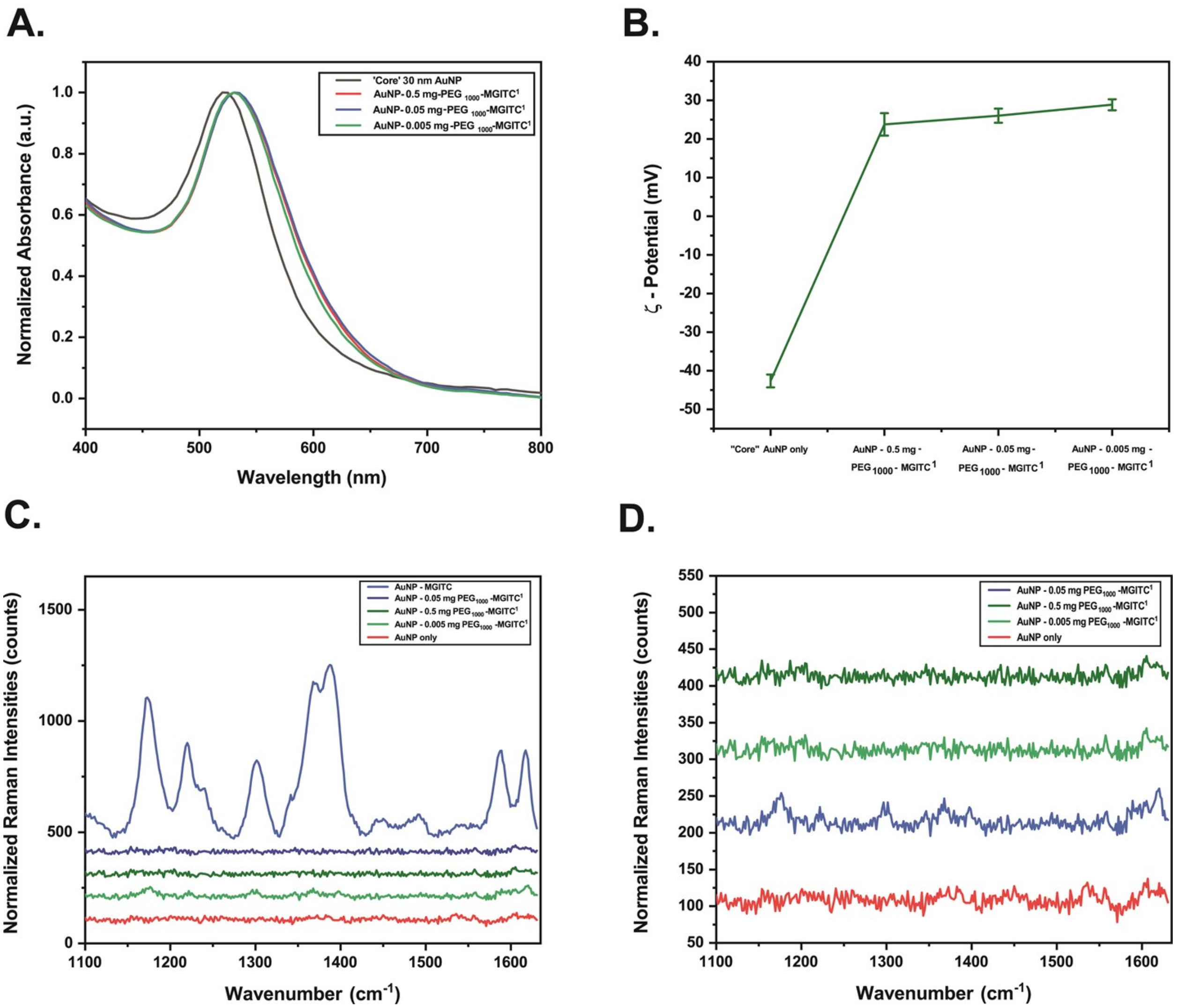

Supplemental Figure S2. Illustrates optimization of various amount of PEG_{1000} deposited on the ~30 nm Core AuNP surface ranging from 1.53 mM (0.5 mg of SH-PEG_{1000}-NH_2 in total volume of 325 µL), 0.153 mM (0.05 mg of SH-PEG_{1000}-NH_2 in total volume of 325 µL), and 0.0153 mM (0.05 mg of SH-PEG_{1000}-NH_2 in total volume of 325 µL) with a fixed amount of 75 µL of 10 µM MGITC. **Fig S2A**. illustrates optical stability after surface modification, **Fig S2B**. illustrates change in surface charge of the AuNPs after deposition of MGITC and PEG_{1000} **Fig S2C.** reports SERS measurement of each respective sample at an excitation of 785 nm. **Fig** S**2D**. depicts each SERS measurement of MGITC and PEG_{1000} modified AuNPs at a smaller scale illustrating no visible Raman peaks.

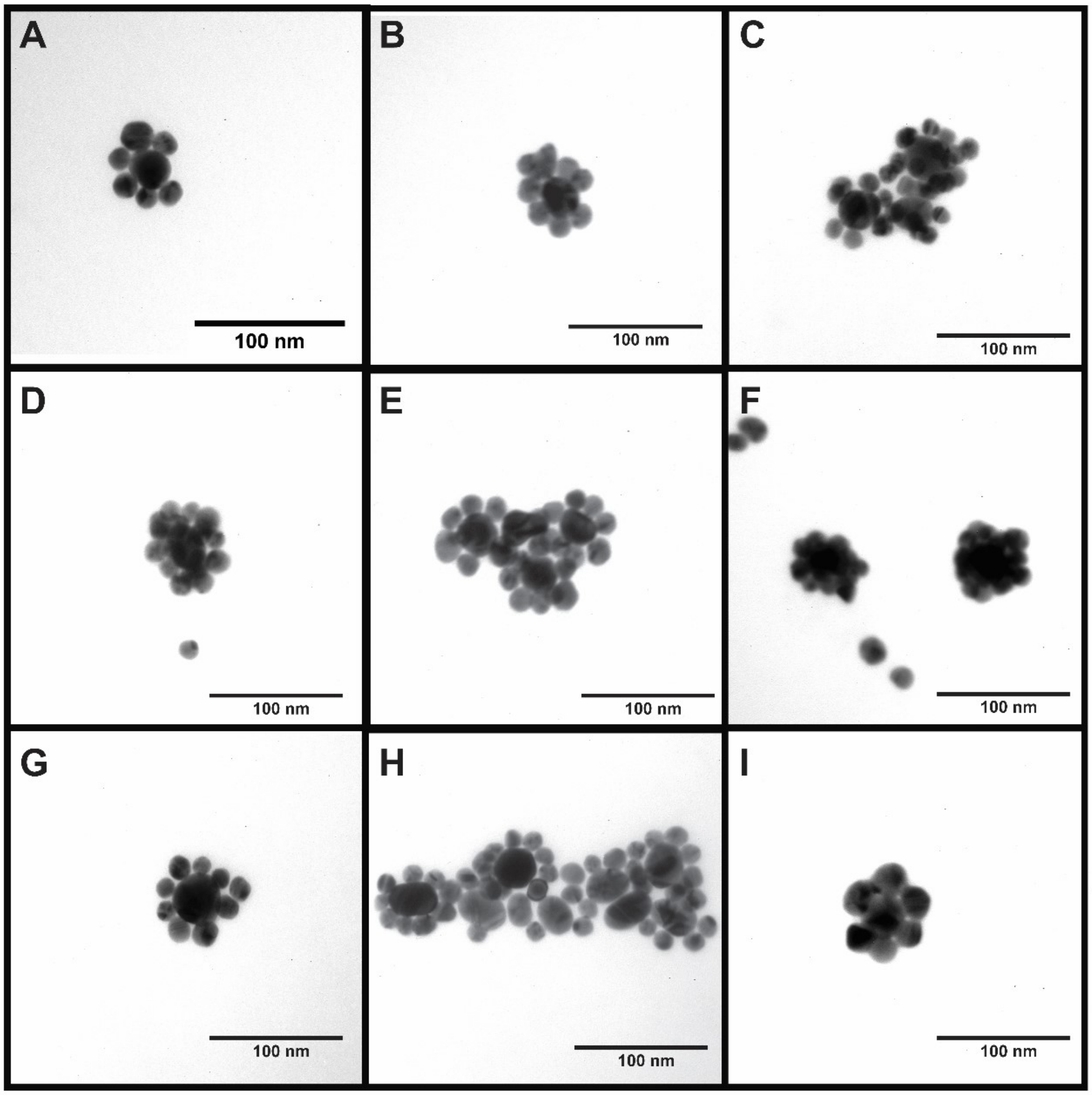

Supplemental Figure S3A-C. Reports TEM images of CSNPs modified with PEG_{1000}-$MGITC^2$ with an average core diameter 30 nm and satellite diameter 18 nm. **Fig S3D-3F.** Au- CSNPs modified with PEG_{2000}-$MGITC^2$. **Fig S3G-I.** CSNPs modified with PEG_{3500}-$MGITC^2$.

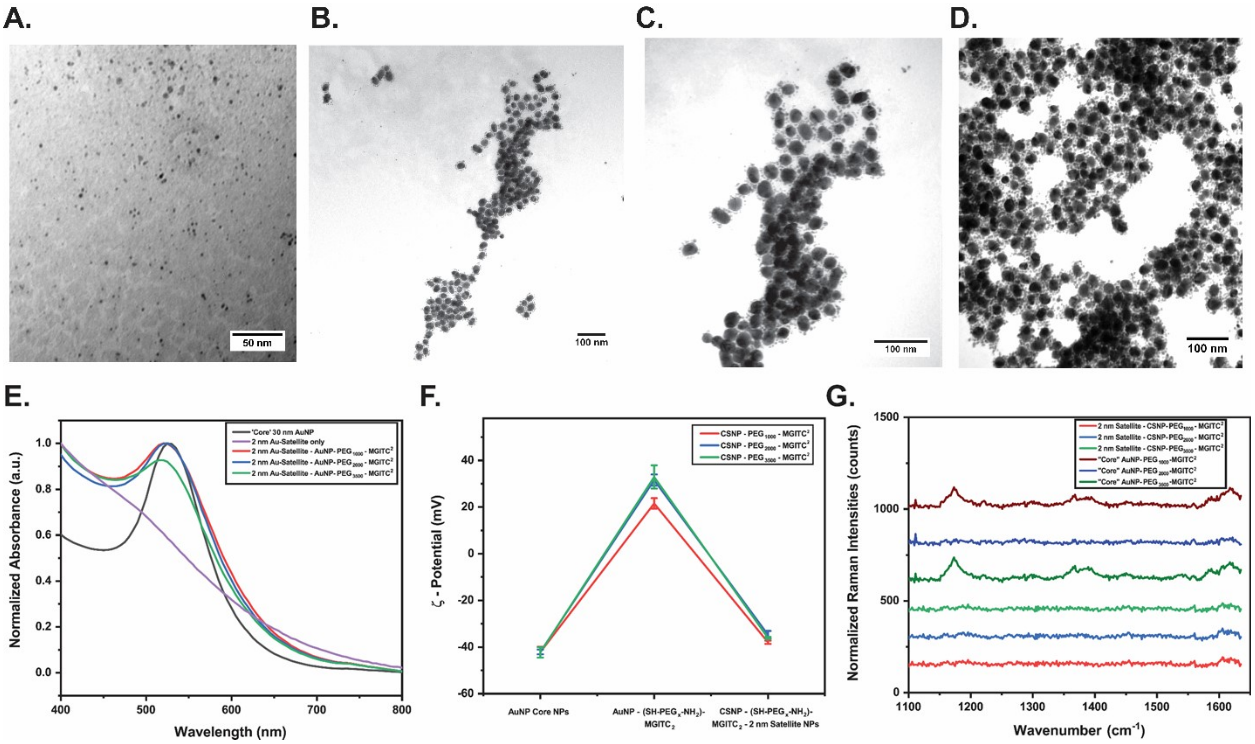

Supplemental Figure S4A. TEM images of synthesized 2 nm AuNP seeds, **Fig S4B.** reporting TEM images of CSNPs modified with 2 nm AuNP seeds, **Fig S4C-D**. illustrating the Fig S4B at a higher magnification confirming the 2 nm AuNP seed coverage. **Fig S4E.** UV-Vis measurement illustrating optical properties of Core AuNPs, 2 nm AuNP seed, CSNPs modified 2nm AuNP seed with each respective CSNPs-PEG$_x$-MGITC^2. **Fig S4F.** ζ-potential measurements of each respective CSNPs-PEG$_x$-MGITC^2 reporting successful surface functionalization at each step. **Fig S4G.** SERS measurement of CSNPs -PEG$_x$-MGITC^2 modified with 2 nm seed with no evident SERS enhancement due to lack of hotspot formation.

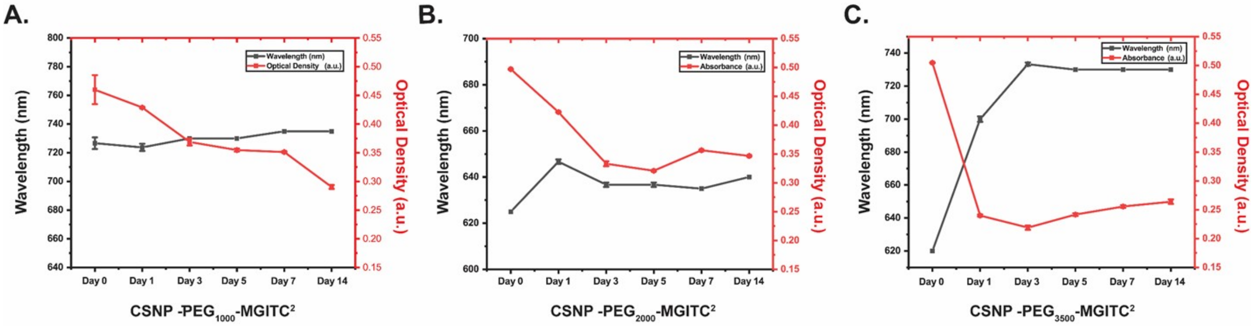

Supplemental Fig S5. Reporting LSPR peak shift and optical density change overtime for CSNPs synthesized with various molecular weights of PEG (1000 Da, 2000 Da, 3500 Da). Additionally, loss of optical density is also reported over 0-14 days representing loss of particles overtime per respective PEG length chain.
